# EpiSUS 25 years: an experience report on advances and legacies in field epidemiology and public health surveillance in Brazil, 2000-2025

**DOI:** 10.1590/S2237-96222025v34e20250532.en

**Published:** 2025-07-28

**Authors:** Magda Machado Saraiva Duarte, Tanna Raposo dos Santos Nagem Morales, Danniely Carolinne Soares da Silva, Maria Isabella Claudino Haslett, Silvio Almeida, Adriana Silveira Cogo, Ana Terra Roque de Araújo, Aroldo José Borges Carneiro, Camila Fernanda dos Santos Santana, Laís Alves Filgueiras Gomes, Luciana Nogueira de Almeida Guimarães, Maria Izabel Lopes, Mariana Sanches de Mello, Ricristhi Gonçalves de Aguiar Gomes, Raquel Proença, Ana Sara Semeão de Souza, Elizabeth David dos Santos, Taynná Vernalha Rocha Almeida, Edenilo Baltazar Barreira

**Affiliations:** ¹ Ministério da Saúde, Departamento de Emergências em Saúde Pública, Brasília, DF, Brazil

**Keywords:** Field Epidemiology, Public Health Surveillance, Unified Health System, Epidemiological Investigation, Experience report., Epidemiología de Campo, Vigilancia en Salud pública, Sistema Único de Salud, Investigación Epidemiológica, Relato de Experiencia

## Abstract

**Objective:** To describe the experience of implementing the Field Epidemiology Training Program for the Brazilian National Health System (*Programa de Treinamento em Epidemiologia Aplicada aos Serviços do Sistema Único de Saúde* - EpiSUS), highlighting key milestones, progress and challenges, and future perspectives for strengthening health surveillance and emergency response in Brazil. **Methods**: This was an experience report based on technical documents, institutional publications of the Program, and key informants. **Results:** EpiSUS was implemented in 2000 with international support and became fully nationalized in 2009. In 2017, the pyramid model began to expand in the country starting from the basic level. Two years later, activities at the intermediate level began, in partnership with the Oswaldo Cruz Foundation, Brasília unit. By early 2025, more than 4,000 professionals had been trained in field epidemiology across the three levels of EpiSUS, most of them at the basic level (3,533), followed by the intermediate (778) and advanced levels (196). **Conclusion:** The outcomes monitored over the years reflect the efforts of management teams and trainees in responding to public health emergencies at the national, state/district, and municipal levels. The implementation of the basic and intermediate levels brought innovations to the training approach. The outputs produced by trainees provide diverse inputs to support timely and evidence-based decision-making.

## Resumen

**Objetivo:** Describir la experiencia de la implementación del Programa de Capacitación en Epidemiología Aplicada a los Servicios del Sistema Único de Salud (EpiSUS), destacando los hitos históricos, los avances y desafíos, y las perspectivas futuras para el fortalecimiento de la vigilancia en salud y la respuesta a emergencias en Brasil. **Métodos:** Se trata de un informe de experiencia basado en documentos técnicos, publicaciones institucionales del Programa e informantes clave. **Resultados:** El EpiSUS se implementó en 2000 con apoyo internacional y alcanzó su nacionalización total en 2009. En 2017, el modelo piramidal comenzó a expandirse en el país a partir del nivel básico y, dos años más tarde, con el inicio de las actividades del nivel intermedio, en colaboración con la Fundación Oswaldo Cruz, unidad de Brasilia. Hasta principios de 2025, se formaron más de 4000 profesionales en epidemiología de campo en los tres niveles del EpiSUS, la mayoría en el nivel básico (3533), seguido de los niveles intermedio (778) y avanzado (196). **Conclusión:** Los resultados observados a lo largo de los años reflejan los esfuerzos de los equipos de gestión y de los profesionales en formación para atender las emergencias de salud pública en los ámbitos nacional, estatal/distrital y municipal. La implementación de los niveles básico e intermedio ha aportado innovaciones a la formación. Los resultados de los productos elaborados por los profesionales en formación generan diversos insumos para la toma de decisiones oportunas y basadas en la evidencia.

**Ethical aspects**

This study did not require approval by a Research Ethics Committee, in accordance with Brazilian National Health Council Resolution No. 466/2012, as it relied exclusively on secondary data sources, such as institutional documents and key informants

## Introduction

Public health emergencies, such as pandemics like COVID-19, highlight the importance of specialized technical knowledge and a qualified public health workforce ^1^. Strengthening health systems-particularly their workforce-is essential to fulfilling the core capacities required under the International Health Regulations ^2^. An effective response requires an adequate number of qualified professionals, equitably distributed across levels of care, surveillance activities, and strategic sectors ^3^.

Field epidemiology is critical for public health surveillance, as it contributes to health protection, infectious disease control, and the strengthening of global public health. ^4^. In Brazil, its relevance is amplified within the framework of the Brazilian National Health System (*Sistema Único de Saúde* - SUS), as it enables timely, evidence-based responses to outbreaks and public health emergencies.

The Field Epidemiology Training Program (FETP) aims to build capacity in the public health workforce, with an emphasis on developing core competencies in field epidemiology, health surveillance, and response to emergencies and public health events ^5^. In Brazil, the FETP was established in 2000 under the name Field Epidemiology Training Program applied to the Unified Health System Services (*Programa de Treinamento em Epidemiologia Aplicada aos Serviços do Sistema Único de Saúde* - EpiSUS) to strengthen the structure of the national public health surveillance system.

A key feature of the FETP is its service-based training model, with approximately 75.0% of the training conducted in the field, within health services across different levels of management ^6^. Initially restricted to the advanced level-lasting 24 months and requiring full-time commitment-the Program was expanded in recent years to include the basic (or frontline) level, lasting three months, and the intermediate level, lasting between nine and twelve months ^5^ ^,^ ^6^.

The global expansion of FETPs has enhanced the capacity to respond to public health threats in various contexts. Immediate impacts can be seen in surveillance activities, especially in outbreak investigations conducted by trainees. These actions help to interrupt chains of disease transmission and generate recommendations for improving services and reinforcing public health actions ^1^ ^,^ ^6^ ^,^ ^7^.

EpiSUS was developed in response to the needs and characteristics of SUS, based on the population’s epidemiological profile and health needs. Its primary focus is to enhance preparedness and response capacity for public health emergencies at the federal, state/district, and municipal levels ^8^ ^-^ ^10^.

Since its inception, the Program has played a strategic role in training field epidemiologists, strengthening the public health workforce, and improving surveillance services, particularly in ensuring a timely response to outbreaks and epidemics in diverse population settings. In this context, the present paper aims to describe the experience of implementing EpiSUS, highlighting its historical milestones, achievements, and challenges, as well as future perspectives for strengthening health surveillance and preparedness and response to public health emergencies in Brazil.

## Methods

This was an experience report describing the advances and legacy of EpiSUS implementation in Brazil between 2000 and 2025.

The data sources included technical documents and institutional publications produced by the Program itself and by other technical departments within the Brazilian Ministry of Health, as well as consultations with key informants directly involved in its design, management, and implementation over the years.

The information was selected and organized by thematic axis to reflect the key milestones in the trajectory of EpiSUS, as identified by the authors: implementation of different training levels, program management within the tripartite structure, activities in response to public health emergencies, and the impacts observed in health surveillance. 

Among the methodological limitations of this study, the most notable were the difficulty in identifying all institutional documents related to the Program and the scarcity of systematized records from its early years of operation.

## Results

In 1998, the National Epidemiology Center-later restructured as the Health and Environment Surveillance Secretariat of the Brazilian Ministry of Health (*Secretaria de Vigilância em Saúde e Ambiente do Ministério da Saúde*)-was notified of an outbreak of kidney disease of unknown origin in Nova Serrana, Minas Gerais. At the time, due to difficulties in identifying the source of infection and controlling the situation, the Ministry of Health requested support from the Centers for Disease Control and Prevention (CDC) through the Epidemic Intelligence Service (EIS) and the U.S. Field Epidemiology Training Program (FETP). 

The investigation was conducted by EIS professionals in collaboration with technicians from the affected municipality, the state of Minas Gerais, and the Ministry of Health. This event highlighted an important gap in the training of health professionals to work in field epidemiology and respond to public health emergencies in Brazil. As a result, the Brazilian Ministry of Health, in technical cooperation with the U.S. CDC, initiated the implementation of the Brazilian FETP, EpiSUS, at the advanced level.

**Figure 1:**
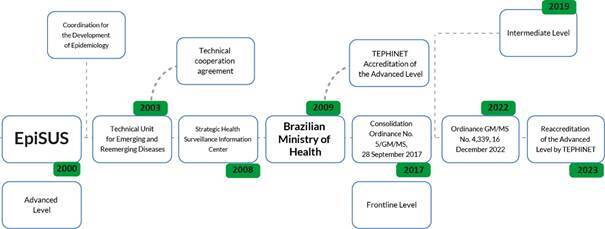
Timeline with the main institutional and regulatory milestones in the implementation and consolidation of the Field Epidemiology Training Program for the Brazilian National Health System (EpiSUS). Brazil, 2000-2023

During the initial implementation phase (2000-2003), the Program was managed by the Coordination for the Development of Epidemiology and received technical support from two CDC consultants. With the establishment of the Health Surveillance Secretariat in 2003, EpiSUS became part of the Technical Unit for Emerging and Reemerging Diseases (*Unidade Técnica de Doenças Emergentes e Reemergentes*), continuing under the technical guidance of a CDC consultant (Figure 1).

As of July 2008, EpiSUS was incorporated into the organizational structure of the Strategic Health Surveillance Information Center. Later that year, the Brazilian Ministry of Health and the National Council for Scientific and Technological Development (*Conselho Nacional de Desenvolvimento Científico e Tecnológico* - CNPq) signed a technical cooperation agreement, which included a decentralized funding mechanism that enabled payment of training stipends for advanced-level trainees. This partnership contributed to the institutionalization of EpiSUS and the continuity of its activities. In 2009, EpiSUS became managed exclusively by the Brazilian Ministry of Health, with qualified professionals trained by the initiative itself (Figure 1). 

Initially offered only at the advanced level, EpiSUS was formally regulated by Consolidation Ordinance/GM/MS No. 5 of 28 September 2017. This regulation was later updated by Ordinance GM/MS No. 4.339 of 16 December 2022, by which time all three training levels of the Program had been implemented and were operational. The most recent regulation also introduced a tutor training program designed to enhance the instructional skills of professionals involved in delivering EpiSUS content. It is worth noting that the basic and intermediate levels were implemented in 2017 and 2019, respectively.

With the creation of the Department of Public Health Emergencies, established by Decree No. 11,098 of 20 June 2022, EpiSUS was incorporated into its structure.

For nearly two decades, the Program’s efforts were primarily focused on consolidating the advanced training level. Following the development of the three-level structure (basic, intermediate, and advanced), EpiSUS adopted various training formats designed to meet the specific demands of the municipal, state/district, and national levels. 

The advanced level is characterized by intensive field immersion, with trainees working closely with technical teams from the Program and the Health and Environment Surveillance Secretariat. Throughout the two-year training, professionals-working under a full-time commitment-are based in Brasília and deployed to conduct field activities as needed. Although the Program’s development is historically associated with the advanced level, it is equally important to acknowledge the progress and impact of the other training levels. 

In October 2016, in the context of arbovirus outbreaks in Brazil, particularly the Zika virus epidemic, the EpiSUS team hosted representatives from the Training Programs in Epidemiology and Public Health Interventions Network (TEPHINET). The goal was to explore new strategies to strengthen surveillance and response activities. As a result, a letter of intent was signed, and in February 2017, the EpiSUS-Frontline pilot project was launched. 

The Basic level (or frontline) aims to enhance the technical capacity of local health surveillance professionals in activities such as disease and condition reporting, outbreak investigations, and health data communication. Its implementation has strengthened the autonomy and responsiveness of states and municipalities. As of 2025, EpiSUS-Frontline has been decentralized to subnational governments to further expand its national reach.

The intermediate level was implemented in 2019 through a partnership between the Department of Environmental and Occupational Health and Public Health Emergencies (*Departamento de Saúde Ambiental, do Trabalhador e Vigilância das Emergências em Saúde Pública*)-where the Program was then located-and the Epidemiology and Health Surveillance Center at the Brasília campus of the Oswaldo Cruz Foundation (*Núcleo de Epidemiologia e Vigilância em Saúde da Fundação Oswaldo Cruz*). Given its workload, content, and complexity, this level was structured as a *lato sensu* graduate program, awarding participants the academic title of Specialist in Field Epidemiology. Courses are offered across the country’s macro-regions through partnerships with certifying academic institutions, which play a key role in implementing the training modules.

**Figure 2:**
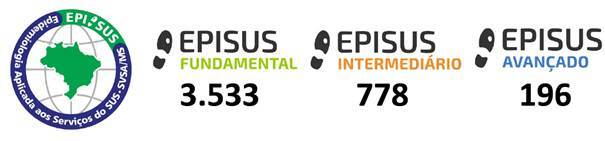
Number of graduates from the Field Epidemiology Training Program for the Brazilian National Health System (EpiSUS) by level of education (basic, intermediate, and advanced). Brazil, January 2000 to April 2025 (n=4,507)

Both the basic and intermediate levels are delivered alongside participants’ routine professional duties, enabling professionals to train while remaining in service. By 2025, more than 4,000 professionals had been trained in field epidemiology through EpiSUS across all three levels: 3,533 at the basic level, 778 at the intermediate level, and 196 at the advanced level (Figure 2).

Since its launch in 2017, EpiSUS-Frontline has trained over 100 cohorts, with presence in every Brazilian state. As of this report, the Program had reached 60.0% of health macro-regions and 21.0% of municipalities with more than 100,000 inhabitants, totaling 3,533 professionals trained at this level. Despite increasing diversity in the professional backgrounds of participants, nursing remained the most common background (Table 1).

**Table 1:** Number of professionals graduating Public health of basic training and their area of specialization. Brazil, 2017-2024 (n=3,533)

Area of specialization	2017	2018	2019	2020	2021	2022	2023	2024
Biomedicine	6	11	12	-	-	15	5	11
**Area of specialization**	2017	2018	2019	2020	2021	2022	2023	2024
Biomedicine	6	11	12	-	-	15	5	11
Biotechnology	-	-	-	-	-	-	-	1
Biological Sciences	15	42	49	1	2	40	23	10
Physical Education	-	-	-	-	-	-	3	-
Nursing	131	439	314	75	61	402	161	179
Pharmacy	7	24	20	6	1	33	19	14
Physiotherapy	4	17	9	5	3	14	5	2
Phonoaudiology	-	4	1	1	-	2	-	2
Medicine	1	10	19	-	-	12	8	11
Veterinary Medicine	8	24	43	3	1	24	18	16
Nutrition	-	-	-	-	2	12	5	3
Dentistry	-	-	-	-	-	17	4	11
Psichology	-	-	-	-	1	10	6	4
Public health	-	-	-	-	1	35	9	10
Technician	13	28	27	1	-	39	16	17
Other training area^a^	21	138	96	11	14	62	28	36
No information	-	-	-	-	-	278	132	41
Total	206	737	590	103	86	995	442	374

**Table 2:** Number of graduates per year and area of specialization. Brazil, january 2019 to April 2025 (n=778)

Area of specialization	2019	2021	2023	2024	2025
Business Administration	-	3	-	1	-
Systems Analysis and Development	-	2	-	-	1
Anthropology	-	-	1	-	-
Biomedicine	-	19	-	-	-
Biological Sciences	4	43	6	4	-
Law	-	1	1	1	-
Physical Education	1	2	-	-	-
Nursing	7	305	16	34	18
Fisheries Engineering	-	-	-	1	-
Statistics	-	4	-	-	-
Pharmacy	2	26	1	2	2
Physical Therapy	-	19	-	-	5
Geography	-	4	-	-	-
Medicine	-	16	-	-	-
Veterinary Medicine	3	29	-	2	5
Nutrition	-	15	2	-	-
Dentistry	2	22	-	-	1
Psychology	-	7	-	-	-
Public health	-	11	-	-	-
Social Work	-	7	1	2	-

a Engineering, Economics, Education, Speech Therapy, Environmental Sanitation, and others.

**Table 3:** Number of cohorts, graduates, and professionals in advanced training according to year of admission and areas of specialization. Brazil, January 2000 to March 2025 (n=217)

**Year of admission**	**2000**	**2001**	**2004**	**2005**	**2006**	**2007**	**2008**	**2009**	**2010**	**2011**	**2012**	**2014**	**2016**	**2017**	**2018**	**2019**	**2021**	**2022**	**2023**	**2024**	**2025**
**Cohort**	1	2	3	4	5	6	7	8	9	10	11	12	13	14	15	16	17	18	19	20^a^	21^a^
**Areas of specialization**																					
Nursing	4	5	1	6	1	2	2	3	3	3	6	4	1	7	2	5	4	4	4	2	5
Veterinary Medicine	1	4	3	2	7	2	3	3	3	2	-	2	-	1	2	-	2	5	2	1	1
Biological Sciences	-	-	4	2	1	3	-	2	-	2	3	2	1	2	1	1	-	-	2	1	1
Medicine	6	1	2	2	-	-	-	2	1	-	-	-	-	1	2	-	-	-	-	2	-
Pharmacy	-	-	1	1	2	1	-	1	-	-	-	-	1	2	-	1	2	1	-	-	-
Biomedicine	-	-	-	-	2	1	-	-	-	-	-	-	-	-	-	1	-	3	2	2	-
Dentistry	-	-	-	-	-	1	2	1	1	1	-	-	-	-	1	-	-	-	1	1	-
Collective Health	-	-	-	-	-	-	-	-	-	-	-	-	-	-	-	2	1	-	1	1	2
Nutrition	-	-	-	-	-	-	-	2	-	-	1	-	-	-	-	1	1	-			1
Psychology	-	-	-	-	-	-	-	-	-	-	-	-	-	-	-	-	-	1	-	-	1
Physiotherapy	-	-	-	-	-	-	-	-	-	-	-	-	-	-	-	-	2	-	-	-	-
Total	11	10	11	13	13	10	7	14	8	8	10	8	3	13	8	11	12	14	12	10	11

a Cohorts in progress, number subject to change

Since 2019, EpiSUS-Intermediate has trained 778 professionals specializing in field epidemiology for the SUS. Graduates have conducted over 750 evaluations of health surveillance systems, supported outbreak investigations, planned health surveys to assess vaccination coverage and factors associated with vaccine hesitancy, studied health practices, including those related to sexually transmitted infections, and investigated contexts involving emerging diseases in Brazil, such as COVID-19 and Oropouche fever. The most common undergraduate degrees among graduates at this level were nursing and biology (Table 2).

Between 2000 and 2025, 21 cohorts of advanced-level trainees were selected, with 19 having completed their training by 2025 and two cohorts still in progress-the 20th (2024-2026) and the 21st (2025-2027) (Table 3). Over time, as the Program and training structure became more consolidated, professionals from diverse fields of knowledge began to join the selected cohorts. While only three academic degrees were represented in 2000, this number increased to 14 by 2017, significantly expanding the professional diversity at the advanced level. These professionals supported more than 500 field investigations across the national territory and abroad.

In 2018, EpiSUS received the Director’s Award for Excellence in Epidemiology and Public Health Response from the CDC, in recognition of the Program’s significant contributions to successful public health emergency responses. In the same year, the Program’s advanced level received TEPHINET accreditation. It is a periodic evaluation conducted by the TEPHINET, which ensures that programs meet high standards of quality.

In 2023, the Program underwent re-accreditation at the advanced level and was recognized with distinction. This designation indicates that the Program exceeded the minimum requirements and demonstrated excellence in areas such as pedagogical innovation, notable impact on public health, and high-quality training of field epidemiologists. Following the re-accreditation process, EpiSUS also received commendations for its contributions in “providing evidence for decision-making” and “public health emergency response.”

## Discussion

The findings of this experience report demonstrate that EpiSUS is one of the most important public policies for training in field epidemiology, with a significant impact on strengthening health surveillance and the response to public health emergencies in Brazil. The implementation of the Program’s three levels has resulted in the training of over 4,000 professionals from various disciplines, expanding both the technical reach and diversity within the SUS. The Program’s decentralized structure, combined with the integration of technical training and in-service practice, has facilitated the production of applied evidence, improved surveillance systems, and strengthened local response capacities. Furthermore, progress has been observed in the institutionalization of the Program, the adoption of equity policies, and recognition by national and international public health institutions.

The EpiSUS experience should be understood in light of the political and structural transformations within the Brazilian health system. Establishing SUS and consolidating the health surveillance model required training qualified human resources capable of working in diverse and decentralized contexts. ^11^ ^,^ ^12^. Since then, the Program has contributed to strengthening health surveillance through practice-oriented technical training adapted to the specificities of SUS, without overlapping with existing academic epidemiology programs in the country ^9^.

Based on TEPHINET guidelines ^13^, which recommend a pyramidal structure for FETPs, operating all three EpiSUS levels in Brazil is challenging due to the country’s territorial expanse and regional particularities. The adoption of a decentralized, less hierarchical teaching-learning model tailored to the realities of professional practice has promoted greater engagement by subnational entities and enhanced coordinated action among levels of government in responding to public health emergencies.

The inclusion of the basic and intermediate levels in training has introduced relevant pedagogical innovations. The trainees’ products have proven helpful in improving local health surveillance activities and supporting timely decision-making. Outbreak investigations and other public health events conducted by EpiSUS trainees, along with the technical products developed during training, have generated relevant evidence to inform decision-making within the SUS. These contributions have led to concrete improvements in health surveillance practices, including the inclusion of new vaccines in the national immunization schedule, the enhancement of surveillance systems, the control and mitigation of outbreaks, and a reduction in deaths and adverse outcomes ^10^ ^,^ ^14^. 

Equity-related considerations have been incorporated into Program management. Since 2023, affirmative action quotas have been implemented in the intermediate and advanced levels, representing a milestone in promoting equity, diversity, and social justice in access to public health training ^15^. Moreover, the strong presence of women in technical and leadership roles within the Program contributes to reducing gender disparities in field epidemiology and within SUS ^10^ ^,^ ^16^.

In 2025, the establishment of the Psychosocial Support Unit within EpiSUS-Advanced aligned the Program with SUS principles, reaffirming its commitment to equity and comprehensive care. This strategy aligns with the Brazilian National Mental Health Policy (*Política Nacional de Saúde Mental*) and the Brazilian National Policy on Continuing Education in Health (*Política Nacional de Educação Permanente em Saúde*), reinforcing the understanding that psychosocial care is a key component of training and work processes in public health. ^17^ ^,^ ^18^.

The sustainability of the Program is an essential component of its institutionalization. The incorporation of funding into the National Health Fund in 2009, along with the implementation of a partnership with the National Council for Scientific and Technological Development (CNPq), ensured the continued operation of the advanced level over the years ^16^ ^,^ ^19^. The provision of scholarships for full-time dedication and recognition, as outlined in ministerial regulations, has contributed to the stability and legitimacy of the training offered ^13^.

By receiving external recognition through TEPHINET accreditation and re-accreditation, as well as the CDC award in 2018, the Program has confirmed its international relevance and excellence ^19^ ^,^ ^20^. Participation in networks such as RedSur (South American Field Epidemiology Network) and Lusófona (Network of Field Epidemiology Programs of Portuguese-speaking Countries) has expanded technical cooperation among programs, positioning EpiSUS as a reference in Latin America and among Portuguese-speaking countries.

This study has limitations inherent to its design. The use of key informants was subject to recall bias. The lack of systematized records during the early years of the Program posed challenges to a more comprehensive reconstruction of its history. Lastly, the reliance on institutional sources and the authors’ involvement with the Program may have introduced interpretation bias. Nevertheless, the analyzed data allowed for the identification of milestones in the initiative’s development.

The findings of this study reinforce the foundational role of EpiSUS in training qualified professionals for health surveillance and for responding to public health emergencies in Brazil. The Program’s trajectory demonstrates progress in strengthening a technically skilled workforce, generating evidence for public health decision-making, and building an articulated network of field epidemiologists embedded across different levels of the SUS. 

Given the increasing complexity of 21st-century health challenges-driven by emerging and reemerging diseases, pandemics, climate change, and new dynamics of population mobility-there is a growing need to institutionalize policies that support the effective integration of graduates into surveillance structures. In this regard, it is crucial to establish specific career paths for epidemiologists and public health emergency professionals in Brazil, as well as to maintain and expand the Program’s training levels. The sustainability and ongoing enhancement of EpiSUS depend on institutional commitments that ensure its capacity for adaptation and innovation, reaffirming its role as a strategic instrument for national public health and as an international benchmark in training for applied epidemiology within health services.

## Data Availability

No databases or proprietary analysis codes were used in this research. The analyzed information is publicly available and is described in the Methods section, along with the corresponding citations listed in the References. There is no additional repository to be indicated, nor is a specific database required for this purpose.
