# Supplementary material for: EpiSUS 25 years: an experience report on advances and legacies in field epidemiology and public health surveillance in Brazil, 2000-2025
Source: Epidemiol Serv Saude. 2025 Jul 28;34:e20250532. doi: 10.1590/S2237-96222025v34e20250532.en (PMC12306657; doi:10.1590/S2237-96222025v34e20250532.en)
Supplement: Translation

# Translation: EpiSUS 25 anos: relato de experiência sobre avanços e legados na epidemiologia de campo e vigilância em saúde pública no Brasil, 2000-2025

**DOI:** 10.1590/S2237-96222025v34e20250532.pt

**Authors:** Magda Machado Saraiva Duarte, Tanna Raposo dos Santos Nagem Morales, Danniely Carolinne Soares da Silva, Maria Isabella Claudino Haslett, Silvio Almeida, Adriana Silveira Cogo, Ana Terra Roque de Araújo, Aroldo José Borges Carneiro, Camila Fernanda dos Santos Santana, Laís Alves Filgueiras Gomes, Luciana Nogueira de Almeida Guimarães, Maria Izabel Lopes, Mariana Sanches de Mello, Ricristhi Gonçalves de Aguiar Gomes, Raquel Proença, Ana Sara Semeão de Souza, Elizabeth David dos Santos, Taynná Vernalha Rocha Almeida, Edenilo Baltazar Barreira

**Affiliations:** ¹ Ministério da Saúde, Secretaria de Vigilância em Saúde e Ambiente, Brasília, DF, Brasil

**Keywords:** Epidemiologia de Campo, Vigilância em Saúde Pública, Sistema Único de Saúde, Investigação Epidemiológica, Relato de Experiência.

## Abstract

**Objetivo:** Descrever a experiência da implementação do Programa de Treinamento em Epidemiologia Aplicada aos Serviços do Sistema Único de Saúde (EpiSUS), com destaque para os marcos históricos, avanços e desafios e as perspectivas futuras para o fortalecimento da vigilância em saúde e da resposta às emergências no Brasil. **Métodos:** Tratou-se de relato de experiência baseado em documentos técnicos, publicações institucionais do Programa e informantes-chave. **Resultados:** O EpiSUS foi implementado em 2000 com apoio internacional e alcançou sua total nacionalização em 2009. Em 2017, o modelo piramidal começou a se expandir no país a partir do nível fundamental, e, dois anos mais tarde, com o início das atividades do nível intermediário, em uma parceria com a Fundação Oswaldo Cruz, unidade de Brasília. Até o início de 2025, foram formados mais de 4 mil profissionais em epidemiologia de campo nos três níveis do EpiSUS, sendo a maior parte do nível fundamental (3.533), seguido dos níveis intermediário (778) e avançado (196). **Conclusão:** Os resultados acompanhados ao longo dos anos traduzem os esforços das equipes de gestão e de profissionais em treinamento para atender às emergências em saúde pública nos âmbitos nacional, estadual/distrital e municipal. A implementação dos níveis fundamental e intermediário trouxe inovações para o treinamento. Os resultados dos produtos construídos pelos profissionais em treinamento geram subsídios diversos para a tomada de decisão oportuna e baseada em evidências.

**Aspectos éticos**

Este trabalho não necessitou de apreciação de Comitê de Ética em Pesquisa, conforme a Resolução CNS nº 466/2012, por utilizar exclusivamente fontes de dados secundários, como documentos institucionais e informantes-chave

## Introdução

Emergências em saúde pública, a exemplo de pandemias como a da covid-19, evidenciam a importância do conhecimento técnico especializado e da qualificação da força de trabalho no campo da saúde pública ^1^. O fortalecimento dos sistemas de saúde, em particular no que diz respeito à força de trabalho, é fundamental para o cumprimento das capacidades exigidas pelo Regulamento Sanitário Internacional ^2^. Para assegurar uma resposta eficaz, é necessário o número adequado de profissionais qualificados, distribuídos de maneira equitativa entre os níveis de atenção, nas ações de vigilância e em setores estratégicos ^3^.

A epidemiologia de campo é primordial para a vigilância em saúde pública, pois contribui para a proteção da saúde, o controle de doenças infecciosas e o fortalecimento da saúde pública global ^4^. No Brasil, sua importância se intensifica no âmbito do Sistema Único de Saúde (SUS), ao viabilizar respostas oportunas e baseadas em evidências científicas para conter surtos e emergências emergências em saúde pública.

O modelo Field Epidemiology Training Program (FETP) visa à capacitação da força de trabalho em saúde, com ênfase no desenvolvimento de competências essenciais em epidemiologia de campo, vigilância em saúde e resposta a emergências e eventos de interesse em saúde pública ^5^. No Brasil, o FETP recebeu o nome de Programa de Treinamento em Epidemiologia Aplicada aos Serviços do Sistema Único de Saúde (EpiSUS) e foi implementado em 2000, a fim de fortalecer a estrutura do sistema nacional de vigilância em saúde pública.

A principal característica do FETP é o treinamento em serviço, com cerca de 75,0% da formação realizada em campo, em serviços de saúde nos distintos níveis de gestão ^6^. Inicialmente restrito ao nível avançado - com duração de 24 meses e dedicação exclusiva -, o Programa foi ampliado, nos últimos anos, para incluir os níveis fundamental (ou linha de frente), com duração de três meses, e intermediário, com duração de nove a 12 meses ^5^ ^,^ ^6^.

A expansão dos FETP em nível global tem fortalecido a capacidade de resposta a riscos em saúde pública em diversos contextos. Observam-se impactos imediatos nas ações de vigilância, especialmente nas investigações epidemiológicas de surtos conduzidas por profissionais em treinamento. Tais ações contribuem para a interrupção de cadeias de transmissão de doenças, além de gerarem recomendações para aprimorar os serviços e reformular e fortalecer as ações de saúde pública ^1^ ^,^ ^6^ ^,^ ^7^.

O EpiSUS foi desenvolvido conforme as necessidades e características do SUS, com base no perfil epidemiológico da população e nas demandas em saúde. Seu foco é o aprimoramento da capacidade de preparação e resposta a emergências em saúde pública nos níveis federal, estadual/distrital e municipal ^8^ ^-^ ^10^.

Desde a sua criação, o Programa atua de forma estratégica na formação de profissionais em epidemiologia de campo, na qualificação da força de trabalho e no aprimoramento dos serviços de vigilância em saúde, com destaque para a resposta oportuna a surtos e epidemias em diferentes contextos populacionais. Nesse sentido, este artigo objetivou descrever a experiência da implementação do EpiSUS, com destaque para os marcos históricos, avanços e desafios e as perspectivas futuras para o fortalecimento da vigilância em saúde e da preparação e resposta às emergências no Brasil.

## Métodos

Tratou-se de relato de experiência que descreveu os avanços e legados da implementação do EpiSUS no Brasil entre 2000 e 2025.

Como fontes de dados, foram utilizados documentos técnicos e publicações institucionais produzidos pelo próprio Programa e por outras áreas técnicas do Ministério da Saúde, além de consultas a informantes-chave diretamente envolvidos em sua concepção, gestão e implementação ao longo dos anos.

As informações foram selecionadas e organizadas por eixos temáticos, buscando refletir os principais marcos da trajetória do EpiSUS elencados pelos autores: a implementação dos diferentes níveis de formação; a gestão do Programa no âmbito tripartite; a atuação nas emergências em saúde pública; e os impactos observados na vigilância em saúde. 

Entre as possíveis limitações metodológicas do estudo, destacaram-se a dificuldade de identificar todos os documentos institucionais sobre o Programa e a escassez de registros sistematizados referentes aos primeiros anos de sua operação.

## Resultados

Em 1998, o Centro Nacional de Epidemiologia, estrutura que originou a Secretaria de Vigilância em Saúde e Ambiente do Ministério da Saúde, foi informado sobre a ocorrência do surto de doença renal de origem desconhecida em Nova Serrana, Minas Gerais. Na ocasião, devido a dificuldades no processo de identificação da fonte de infecção e controle da situação, o Ministério da Saúde solicitou apoio aos Centros de Controle e Prevenção de Doenças (CDC), por meio do Epidemic Intelligence Service (EIS)*,* FETP dos Estados Unidos. 

A investigação foi realizada pelos profissionais do EIS, em parceria com técnicos do município afetado, do estado mineiro e do Ministério da Saúde. Esse evento evidenciou importante lacuna na formação de profissionais de saúde para atuação em epidemiologia de campo e resposta às emergências em saúde pública no território nacional. Sendo assim, o Ministério da Saúde, em cooperação técnica com o CDC estadunidense, iniciou a implantação do FETP no Brasil, o EpiSUS, no nível avançado.

Nos primeiros anos de sua implantação (2000-2003), o Programa foi gerenciado pela Coordenação de Apoio ao Desenvolvimento da Epidemiologia e contou com a expertise técnica de dois consultores do CDC. Com a criação da Secretaria de Vigilância em Saúde, em 2003, o EpiSUS passou a integrar a Unidade Técnica de Doenças Emergentes e Reemergentes, ainda contando com o suporte técnico de um consultor do CDC (Figura 1).

A partir de julho de 2008, o EpiSUS foi inserido na estrutura organizacional do Centro de Informações Estratégicas em Vigilância em Saúde. No final do mesmo ano, o Ministério da Saúde e o Conselho Nacional de Desenvolvimento Científico e Tecnológico celebraram um acordo de cooperação técnica, por meio de termo de execução descentralizada que viabilizou a transferência de financiamento para o pagamento de bolsas para os profissionais em treinamento no nível avançado. Essa parceria contribuiu para a institucionalização do EpiSUS e para a continuidade de suas atividades. Em 2009, o EpiSUS passou a ser gerido exclusivamente pelo Ministério da Saúde, com atuação de profissionais qualificados pela própria iniciativa (Figura 1). 

**Figura 1:** Linha do tempo com os principais marcos institucionais e normativos da implementação e consolidação do Programa de Treinamento em Epidemiologia Aplicada aos Serviços do Sistema Único de Saúde (EpiSUS). Brasil, 2000-2023

Inicialmente ofertado apenas no nível avançado, o EpiSUS foi normatizado pela Portaria de Consolidação/GM/MS nº 5, de 28 de setembro de 2017. Posteriormente, essa normativa foi atualizada pela Portaria GM/MS nº 4.339, de 16 de dezembro de 2022, ocasião em que os três níveis do Programa já estavam implementados e em funcionamento. A normativa mais recente também implementou a formação de tutores, com o objetivo de qualificar os profissionais responsáveis pelo processo formativo em conteúdo específico voltado ao ensino. Cabe destacar que os níveis fundamental e intermediário foram implementados em 2017 e 2019, respectivamente.

Com a criação do Departamento de Emergências em Saúde Pública, instituído pelo Decreto nº 11.098, de 20 de junho de 2022, o EpiSUS passou a integrá-lo.

Durante quase duas décadas, os esforços do Programa estiveram centrados na consolidação do nível avançado do treinamento. A partir da estruturação dos três níveis de formação (fundamental, intermediário e avançado), o EpiSUS passou a atuar com diferentes formatos de treinamento, concebidos de forma complementar para atender às demandas específicas das esferas municipal, estadual/distrital e nacional. 

O nível avançado é caracterizado pela imersão intensiva no serviço, a partir da convivência dos profissionais em treinamento com as equipes técnicas do Programa e da Secretaria de Vigilância em Saúde e Ambiente. Ao longo dos dois anos do treinamento, os profissionais, em regime de dedicação exclusiva, são designados para atividades de campo a partir de Brasília, onde devem residir durante o período de vigência de suas coortes. Embora a trajetória do Programa esteja ligada ao desenvolvimento do nível avançado, é igualmente relevante conhecer a história e o fortalecimento dos demais níveis de formação. 

Em outubro de 2016, diante do cenário epidemiológico das arboviroses no país, especialmente o enfrentamento da epidemia de infecção pelo Zika vírus, a equipe do EpiSUS recebeu no Brasil representantes da Training Programs in Epidemiology and Health Interventions Network (TEPHINET). O principal objetivo foi discutir novas estratégias que possibilitassem o fortalecimento das ações de vigilância e a resposta à epidemia e suas consequências. Como resultado, foi celebrada uma carta de intenções, e, em fevereiro de 2017, o projeto-piloto do EpiSUS-Fundamental teve início. 

O nível fundamental objetiva aprimorar a capacidade técnica dos profissionais que atuam nas ações locais de vigilância em saúde, incluindo atividades como notificação de agravos e doenças, investigação de surtos e comunicação de dados em saúde. Sua implementação representa o fortalecimento da autonomia e a capacidade de resposta dos estados e municípios brasileiros e, por isso, em 2025, EpiSUS-Fundamental é descentralizado para os entes federativos, a fim de expandir o alcance em todo o território nacional.

O nível intermediário foi implementado em 2019, por meio de parceria firmada entre o Departamento de Saúde Ambiental, do Trabalhador e Vigilância das Emergências em Saúde Pública, onde o Programa estava alocado à época, e o Núcleo de Epidemiologia e Vigilância em Saúde da Fundação Oswaldo Cruz, unidade de Brasília. Em face de carga horária, conteúdo e complexidade, esse nível foi estruturado como um curso de pós-graduação lato sensu, oferecendo aos concluintes o título acadêmico de especialista em epidemiologia de campo. As ofertas se concentram nas macrorregiões do país, por meio de parcerias com instituições de ensino certificadoras, que desempenham papel fundamental na operacionalização dos módulos e das atividades formativas.

Os níveis fundamental e intermediário são conduzidos de forma integrada ao exercício profissional, o que viabiliza a participação dos profissionais de maneira concomitante ao serviço. Até 2025, foram formados mais de 4 mil profissionais em epidemiologia de campo nos três níveis do EpiSUS, sendo a maior parte do nível fundamental (3.533), seguida dos níveis intermediário (778) e avançado (196) (Figura 2).

Desde a sua implementação em 2017, o EpiSUS-Fundamental formou mais de 100 coortes, com atuação em todas as unidades federativas do Brasil. Até a publicação deste relato, o nível alcançou 60,0% das macrorregiões de saúde, totalizando 21,0% dos municípios com mais de 100 mil habitantes, somando 3.533 profissionais formados nesse nível. Apesar do aumento da diversidade nas formações profissionais dos participantes do treinamento, a enfermagem permaneceu como a formação mais frequente (Tabela 1).

**Figura 2:** Número de egressos do Programa de Treinamento em Epidemiologia Aplicada aos Serviços do Sistema Único de Saúde (EpiSUS) por nível de formação (fundamental, intermediário e avançado). Brasil, janeiro de 2000 a abril de 2025 (n=4.507)

**Tabela 1:** Número de profissionais egressos do nível fundamental segundo ano e área de formação. Brasil, 2017-2024 (n=3.533)

**Área de formação**	**2017**	**2018**	**2019**	**2020**	**2021**	**2022**	**2023**	**2024**
Biomedicina	6	11	12	-	-	15	5	11
Biotecnologia	-	-	-	-	-	-	-	1
Ciências biológicas	15	42	49	1	2	40	23	10
Educação física	-	-	-	-	-	-	3	-
Enfermagem	131	439	314	75	61	402	161	179
Farmácia	7	24	20	6	1	33	19	14
Fisioterapia	4	17	9	5	3	14	5	2
Fonoaudiologia	-	4	1	1	-	2	-	2
Medicina	1	10	19	-	-	12	8	11
Medicina veterinária	8	24	43	3	1	24	18	16
Nutrição	-	-	-	-	2	12	5	3
Odontologia	-	-	-	-	-	17	4	11
Psicologia	-	-	-	-	1	10	6	4
Saúde coletiva	-	-	-	-	1	35	9	10

a Administração, biblioteconomia, ciências sociais, direito, educação física, engenharia, jornalismo, letras, oceanografia, pedagogia e terapia ocupacional.

**Tabela 2:** Número de profissionais egressos do nível intermediário segundo ano e área de formação. Brasil, 2019 a abril de 2025 (n=778)

**Área de formação**	**2019**	**2021**	**2023**	**2024**	**2025**
Administração	-	3	-	1	-
Análise e desenvolvimento de sistemas	-	2	-	-	1
Antropologia	-	-	1	-	-
Biomedicina	-	19	-	-	-
Ciências biológicas	4	43	6	4	-
Direito	-	1	1	1	-
Educação física	1	2	-	-	-
Enfermagem	7	305	16	34	18
Engenharia de pesca	-	-	-	1	-
Estatística	-	4	-	-	-
Farmácia	2	26	1	2	2
Fisioterapia	-	19	-	-	5
Geografia	-	4	-	-	-
Medicina	-	16	-	-	-
Medicina veterinária	3	29	-	2	5
Nutrição	-	15	2	-	-
Odontologia	2	22	-	-	1
Psicologia	-	7	-	-	-
Saúde coletiva	-	11	-	-	-
Serviço social	-	7	1	2	-
Tecnologia em gestão ambiental	-	-	-	-	1
Tecnologia em processos gerenciais	-	-	-	1	-
Terapia ocupacional	-	5	-	-	-
Outras formações^a^	-	110	-	-	-
**Total**	19	650	28	48	33

a Engenharias, economia, pedagogia, fonoaudiologia, saneamento ambiental e outros.

**Tabela 3:** Número de coortes, egressos e profissionais em treinamento do nível avançado segundo ano de entrada e áreas de formação. Brasil, janeiro de 2000 a março de 2025 (n=217)

**Ano de início**	**2000**	**2001**	**2004**	**2005**	**2006**	**2007**	**2008**	**2009**	**2010**	**2011**	**2012**	**2014**	**2016**	**2017**	**2018**	**2019**	**2021**	**2022**	**2023**	**2024**	**2025**
**Coorte**	1	2	3	4	5	6	7	8	9	10	11	12	13	14	15	16	17	18	19	20^a^	21^a^
**Áreas de formação**																					
Enfermagem	4	5	1	6	1	2	2	3	3	3	6	4	1	7	2	5	4	4	4	2	5
Medicina veterinária	1	4	3	2	7	2	3	3	3	2	-	2	-	1	2	-	2	5	2	1	1
Ciências biológicas	-	-	4	2	1	3	-	2	-	2	3	2	1	2	1	1	-	-	2	1	1
Medicina	6	1	2	2	-	-	-	2	1	-	-	-	-	1	2	-	-	-	-	2	-
Farmácia	-	-	1	1	2	1	-	1	-	-	-	-	1	2	-	1	2	1	-	-	-
Biomedicina	-	-	-	-	2	1	-	-	-	-	-	-	-	-	-	1	-	3	2	2	-
Odontologia	-	-	-	-	-	1	2	1	1	1	-	-	-	-	1	-	-	-	1	1	-
Saúde coletiva	-	-	-	-	-	-	-	-	-	-	-	-	-	-	-	2	1	-	1	1	2
Nutrição	-	-	-	-	-	-	-	2	-	-	1	-	-	-	-	1	1	-			1
Psicologia	-	-	-	-	-	-	-	-	-	-	-	-	-	-	-	-	-	1	-	-	1
Fisioterapia	-	-	-	-	-	-	-	-	-	-	-	-	-	-	-	-	2	-	-	-	-
Total	11	10	11	13	13	10	7	14	8	8	10	8	3	13	8	11	12	14	12	10	11

a Coortes em andamento, número sujeito à alteração.

Desde 2019, o EpiSUS-Intermediário capacitou 778 profissionais especialistas em epidemiologia de campo para o SUS. Os egressos realizaram mais de 750 avaliações de sistemas de vigilância em saúde; apoiaram investigações de surtos; planejaram inquéritos de saúde com vistas a conhecer coberturas vacinais e fatores relacionados à hesitação vacinal; estudaram práticas em saúde, incluindo aquelas voltadas às infecções sexualmente transmissíveis; e investigaram cenários relacionados a doenças emergentes no Brasil, como a covid-19 e a febre do Oropouche. A maior frequência quanto às graduações acadêmicas dos egressos desse nível foi de enfermeiros e biólogos (Tabela 2).

Entre 2000 e 2025, 21 coortes de profissionais em treinamento do nível avançado foram selecionadas, chegando em 2025 com a formação de 19 coortes e outras duas em andamento - 20ª, no biênio 2024-2026 e 21ª, em treinamento no período 2025-2027 (Tabela 3). Ao longo do tempo, a partir da estruturação e consolidação do Programa e do treinamento, profissionais de diferentes áreas do conhecimento passaram a compor o perfil de candidatos selecionados, passando de três graduações acadêmicas em 2000 para 14 a partir de 2017, o que ampliou a diversidade profissional do nível. Esses profissionais atenderam a mais de 500 investigações de campo em todo o território nacional e fora dele.

Em 2018, o EpiSUS recebeu o Director’s Award for Excellence in Epidemiology and Public Health Response, concedido pelo CDC em reconhecimento às contribuições significativas do Programa para respostas bem-sucedidas a emergências de saúde pública. No mesmo ano, o nível avançado do Programa obteve o certificado de acreditação da TEPHINET. Essa é uma avaliação periódica feita pela TEPHINET, que visa garantir que os Programas mantenham elevados padrões de qualidade.

Em 2023, o Programa submeteu o nível avançado ao processo de reacreditação, com destaque para o resultado de distinção. Essa categoria mostra que o Programa excedeu os requisitos mínimos e demonstrou excelência em aspectos de interesse, como inovação pedagógica, impacto notável na saúde pública e alta qualidade na formação de seus epidemiologistas. O EpiSUS também recebeu, a partir do processo de reacreditação, mérito nos temas “provimento de evidências para a tomada de decisão” e “resposta às emergências em saúde pública”.

## Discussão

Os achados deste relato de experiência demonstram que o EpiSUS é uma das mais importantes políticas públicas de formação em epidemiologia de campo, com impacto relevante na qualificação da vigilância em saúde e na resposta a emergências em saúde pública no Brasil. A implementação dos três níveis do Programa resultou na formação de mais de 4 mil profissionais de diferentes áreas, ampliando a capilaridade e a diversidade técnica do SUS. A estrutura descentralizada do Programa, aliada à integração entre formação técnica e prática em serviço, tem favorecido a produção de evidências aplicadas, a qualificação de sistemas de vigilância e o fortalecimento da capacidade de resposta local. Além disso, observam-se avanços na institucionalização do Programa, na adoção de políticas de equidade e no reconhecimento por instâncias nacionais e internacionais da saúde pública.

A experiência do EpiSUS deve ser compreendida à luz das transformações políticas e estruturais do sistema de saúde brasileiro. A instituição do SUS e a consolidação do modelo de vigilância em saúde demandaram a formação de recursos humanos qualificados, capazes de atuar em contextos diversos e descentralizados ^11^ ^,^ ^12^. Desde então, o Programa tem contribuído para o fortalecimento da vigilância em saúde, com formação técnica voltada à prática e adaptada às especificidades do SUS, sem se sobrepor aos programas de epidemiologia existentes no país voltados para a formação acadêmica ^9^.

Com base nas diretrizes da TEPHINET ^13^, que orientam a estruturação dos FETP segundo o modelo piramidal, a operacionalização dos três níveis do EpiSUS no Brasil é desafiadora, dadas a extensão territorial e as particularidades regionais do país. A adoção de estratégia de ensino-aprendizagem por um modelo descentralizado, menos hierarquizado e adaptado às necessidades e especificidades do exercício profissional tem permitido maior adesão dos entes federativos e fortalecido a atuação articulada entre os níveis de governo na resposta às emergências em saúde pública.

A inclusão dos níveis fundamental e intermediário no treinamento trouxe inovações pedagógicas relevantes. Os produtos gerados por esses profissionais mostraram-se úteis para a qualificação das ações locais de vigilância em saúde e apoio à tomada de decisão em tempo oportuno. As investigações de surtos e outros eventos de interesse de saúde pública conduzidas por profissionais em formação no EpiSUS, bem como os produtos técnicos desenvolvidos durante o treinamento, têm gerado subsídios relevantes para a tomada de decisão oportuna e baseada em evidências no âmbito do SUS. Esses aportes resultaram em melhorias concretas nas práticas de vigilância em saúde, como a inclusão de vacinas no calendário nacional, o aprimoramento de sistemas de vigilância, o controle e a mitigação de surtos e a redução de óbitos e desfechos adversos ^10^ ^,^ ^14^. 

Aspectos relacionados à equidade foram incorporados à gestão do Programa. A implementação de reservas de vagas afirmativas desde 2023 nos níveis intermediário e avançado representam marco na promoção de equidade, diversidade e justiça social no acesso à formação em saúde pública ^15^. Além disso, a expressiva presença de mulheres em posições técnicas e de liderança do Programa contribui para a redução das desigualdades de gênero na epidemiologia de campo e no SUS ^10^ ^,^ ^16^.

Ao instituir, em 2025, o Núcleo de Apoio Psicossocial do EpiSUS-Avançado, o Programa alinha-se aos princípios do SUS, reforçando o compromisso com a equidade e com a promoção do cuidado integral. Essa estratégia está em consonância com a Política Nacional de Saúde Mental e a Política Nacional de Educação Permanente em Saúde e fortalece a compreensão de que o cuidado psicossocial constitui eixo estruturante dos processos formativos e laborais em saúde pública ^17^ ^,^ ^18^.

A sustentabilidade do Programa constitui dimensão essencial de sua institucionalização. A integração do financiamento ao Fundo Nacional de Saúde em 2009, com a implementação de parceria com o Conselho Nacional de Desenvolvimento Científico e Tecnológico, viabilizou a continuidade do nível avançado ao longo dos anos ^16^ ^,^ ^19^. A concessão de bolsas para dedicação exclusiva e o reconhecimento por normativas ministeriais contribuem para a estabilidade e legitimidade da formação ofertada ^13^.

Ao ser reconhecido externamente por meio da acreditação e da reacreditação concedidas pela TEPHINET e do prêmio do CDC em 2018, o Programa confirmou sua relevância e excelência em âmbito internacional ^19^ ^,^ ^20^. A participação em redes como a RedSur (Rede de Epidemiologia de Campo da América do Sul) e a Lusófona (Rede de Programas de Epidemiologia de Campo de países de língua portuguesa) amplia a cooperação técnica entre Programas e projeta o EpiSUS como referência na América Latina e entre os países de língua portuguesa.

Este estudo apresentou limitações inerentes ao seu delineamento. O uso de informantes-chave esteve sujeito ao viés de memória. A escassez de registros sistematizados nos primeiros anos do Programa impôs dificuldades para a reconstrução histórica de forma mais abrangente. Por fim, a dependência de fontes institucionais e o envolvimento dos autores com o Programa puderam ter introduzido viés de interpretação. Apesar disso, os dados analisados permitiram identificar marcos na evolução da iniciativa.

Os achados deste estudo corroboram o papel estruturante do EpiSUS na formação de profissionais qualificados para a vigilância em saúde e para a resposta a emergências em saúde pública no Brasil. A trajetória desse Programa demonstra avanços na consolidação da força de trabalho tecnicamente capacitada, na geração de evidências para a tomada de decisão em saúde pública e na constituição de rede articulada de epidemiologistas de campo inserida nas diferentes esferas do SUS. 

Diante da crescente complexidade dos desafios sanitários do século XXI, impulsionada por doenças emergentes e reemergentes, pandemias, mudanças climáticas e novas dinâmicas de mobilidade populacional, sobressalta-se a necessidade de institucionalizar políticas que favoreçam a inserção efetiva dos egressos nas estruturas de vigilância. Nesse sentido, destaca-se a importância da criação de carreiras específicas para epidemiologistas e emergencistas no país, bem como da manutenção e expansão dos níveis formativos do Programa. A sustentabilidade e o contínuo aprimoramento do EpiSUS dependem de compromissos institucionais que assegurem sua capacidade de adaptação e inovação, reafirmando-o como instrumento estratégico para a saúde pública nacional e referência internacional na formação em epidemiologia aplicada aos serviços de saúde

## Data Availability

Não foram utilizados bancos de dados ou códigos de análise próprios nesta pesquisa. As informações analisadas são de acesso público e estão descritas na seção Métodos, com as devidas citações incluídas nas Referências. Não há repositório adicional a ser indicado nem banco de dados específico para esse fim.
